# Performance of 18F-DCFPyL PET/CT Imaging in Early Detection of Biochemically Recurrent Prostate Cancer: A Systematic Review and Meta-Analysis

**DOI:** 10.3389/fonc.2021.649171

**Published:** 2021-04-26

**Authors:** Jiale Sun, Yuxin Lin, Xuedong Wei, Jun Ouyang, Yuhua Huang, Zhixin Ling

**Affiliations:** Department of Urology, The First Affiliated Hospital of Soochow University, Suzhou, China

**Keywords:** 18F-DCFPyL, prostate-specific membrane antigen, PET/CT, biochemically recurrent prostate cancer, imaging

## Abstract

**Background:** Prostate-specific membrane antigen (PSMA)-targeted 2-(3-{1-carboxy-5-[(6-[18F] fluoro-pyridine-3-carbonyl)-amino]-pentyl}-ureido)-pentanedioic acid (18F-DCFPyL) positron emission tomography/computed tomography (PET/CT) has shown advantages in primary staging, restaging, and metastasis detection of prostate cancer (PCa). However, little is known about the role of 18F-DCFPyL PET/CT in biochemically recurrent prostate cancer (BRPCa). Hence, we performed a systematic review and meta-analysis to evaluate 18F-DCFPyL PET/CT as first-line imaging modality in early detection of BRPCa.

**Methods:** A comprehensive literature search of PubMed, Web of Science, Embase, and Cochrane Library was conducted until December 2020. The pooled detection rate on a per-person basis and together with 95% confidence interval (CI) was calculated. Furthermore, a prostate-specific antigen (PSA)-stratified performance of detection positivity was obtained to assess the sensitivity of 18F-DCFPyL PET/CT in BRPCa with different PSA levels.

**Results:** A total of nine eligible studies (844 patients) were included in this meta-analysis. The pooled detection rate (DR) of 18F-DCFPyL PET/CT in BRPCa was 81% (95% CI: 76.9–85.1%). The pooled DR was 88.8% for PSA ≥ 0.5 ng/ml (95% CI: 86.2–91.3%) and 47.2% for PSA < 0.5 ng/ml (95% CI: 32.6–61.8%). We also noticed that the regional lymph node was the most common site with local recurrence compared with other sites (45.8%, 95% CI: 42.1–49.6%). Statistical heterogeneity and publication bias were found.

**Conclusion:** The results suggest that 18F-DCFPyL PET/CT has a relatively high detection rate in BRPCa. The results also indicate that imaging with 18F-DCFPyL may exhibit improved sensitivity in BRPCa with increased PSA levels. Considering the publication bias, further large-scale multicenter studies are warranted for validation.

## Introduction

Prostate cancer (PCa) is the most common form of malignant tumor among men in the United States and the second most common cause of cancer-related deaths in aging men (1). After patients received the initial treatments, follow-up strategies including physical examination and prostate-specific antigen (PSA) tests were performed to monitor the progression of the disease (2). Despite the high success rate of the primary treatments, PCa recurrence is relatively common, presenting with a sudden rise or persistently elevated PSA levels. For post radical prostatectomy (RP), biochemical recurrence (BCR) is defined as two consecutive PSA values that are >0.2 ng/ml and rising (3). For post external beam radiation therapy (EBRT), BCR is defined as an increase in the PSA level by 2 ng/ml or more above the nadir (4). It is reported that BCR will occur in ~20–40% of the patients undergoing radical prostatectomy (5–7) and 30–50% of men after EBRT (8). Another study also reported that 13.9% of PCa patients following brachytherapy developed biochemical failure in the first decade of follow-up (9). Although the treatment of men with biochemically recurrent prostate cancer (BRPCa) should be based not only on radiographic characteristics but also on personal clinical, pathologic, and genomic characteristics, and optimal timing of systemic therapy remains controversial (10), the early lesion localization of BRPCa is still essential to define disease distribution that could, in turn, help urologists make further possible clinical decisions including surgery, salvage radiation therapy (RT), androgen deprivation therapy (ADT), or chemotherapy. However, traditional imaging methods such as plain X-ray, CT, MRI, or bone scintigraphy are limited by their low sensitivity while detecting early recurrent disease (11). Developments of imaging techniques may enable urologists to localize recurrence or metastasis sites in BCR patients.

The prostate-specific membrane antigen (PSMA) is specifically highly expressed in the surface of PCa cells (12). Recently, PSMA-targeted positron emission tomography/computed tomography (PET/CT) is increasingly used in PCa diagnostics (13, 14). Moreover, PSMA PET/CT has greater sensitivity and specificity for detecting pelvic nodal or distant metastases than conventional imaging techniques (15). Therefore, PSMA radiopharmaceuticals have been increasingly used to detect small tumor lesions, lymph node, bone, or visceral metastases because of its high sensitivity even at low PSA levels. Several PSMA radioligands such as Gallium-68 (68Ga), Fluorine-18 (18F), Lutetium-177 (177Lu), or Copper-64 (64Cu) are currently available to obtain effective radiotherapeutics for theranostic applications (16–20). 68Ga-PSMA PET/CT was first introduced to predict biochemical recurrence in PCa patients after initial therapy (21, 22). It was reported that lesions suspicious for PCa detected by 68Ga-PSMA PET/CT presented with excellent contrast as early as 1 h post-injection with high detection rates even at low PSA levels (23). However, despite the widespread clinical adoption of this agent, there are some disadvantages related to its short physical half-life (68 min) and decreasing synthesis yields as generators decay. It is also difficult and expensive to comply with good manufacturing practice guidelines, and therefore centralized radiopharmacy production and distribution are constrained (24). By contrast, 18F-labeled PSMA agents seem to have more advantages as they provide a longer half-life, allowing for later facilitating tumor visualization with higher physical spatial resolution (25). The 2-(3-{1-carboxy-5-[(6-[18F] fluoro-pyridine-3-carbonyl)-amino]-pentyl}-ureido)-pentanedioic acid (18F-DCFPyL), as a novel second-generation PSMA agent, binds with higher affinity, thus allowing earlier detection of local recurrence even at a lower PSA level (26, 27). To our knowledge, the value of this kind of radiotracer is still unclear due to the relatively small sizes of the prior studies. Therefore, we performed a meta-analysis to more accurately evaluate the diagnostic performance of 18F-DCFPyL PET/CT in BRPCa patients.

## Materials and Methods

### Search Strategy and Identification of Eligible Studies

Two reviewers (Sun and Lin) searched the online databases PubMed, Web of Science, Embase, and Cochrane Library to identify the relevant articles published until December 2020. The study was reported according to the Preferred Reporting Items for Systematic Reviews and Meta-Analyses (PRISMA) (28), and the following keywords were used: “18F-DCFPyL” OR “2-(3-{1-carboxy-5-[(6-[18F]fluoro-pyridine-3-carbonyl)-amino]-pentyl}-ureido)-pentanedioic acid” AND “Biochemically recurrent prostate cancer.” Articles in all languages were considered relevant for review, and the references of pertinent articles were manually screened and checked as well. All prospective or retrospective studies investigating 18F-DCFPyL PET/CT and BRPCa were included. Exclusion criteria were as follows: (I) case series or case reports; (II) review articles and editorial comments; (III) data incomplete or unclear or unusable with our study or major mistakes; (IV) republished literature; (V) studies performed on a per-lesion basis.

### Data Extraction

Two reviewers (Sun and Lin) extracted the following information independently from each study, and inconsistencies were resolved by discussion until a consensus was obtained. Extracted data included country, study period, study design, sample size, characteristics of participants, technical aspects, and detection rate (DR) of 18F-DCFPyL PET/CT on a per-person basis. Quality Assessment of Diagnostic Accuracy Studies (QUADAS-2) (29) was used for assessing the quality of articles included in this study.

### Statistical Analysis

All statistical analyses were conducted by Stata (version 15; StataCorp, Texas, USA). The heterogeneity between different articles was determined by *I*^2^ index (30). When significant heterogeneity was observed (*I*^2^ > 50%), the random-effect model was applied. Based on our clinical experience, subgroup analysis according to mean/median PSA before 18F-DCFPyL PET/CT scanning was conducted if significant heterogeneity exists. A PSA-stratified performance of detection positivity was obtained to assess the sensitivity of 18F-DCFPyL PET/CT in BRPCa with different PSA levels. Egger's test was conducted to estimate publication bias. All tests with two-sided *P* < 0.05 were considered statistically significant.

## Results

### Characteristics of Included Studies

**Figure 1:**
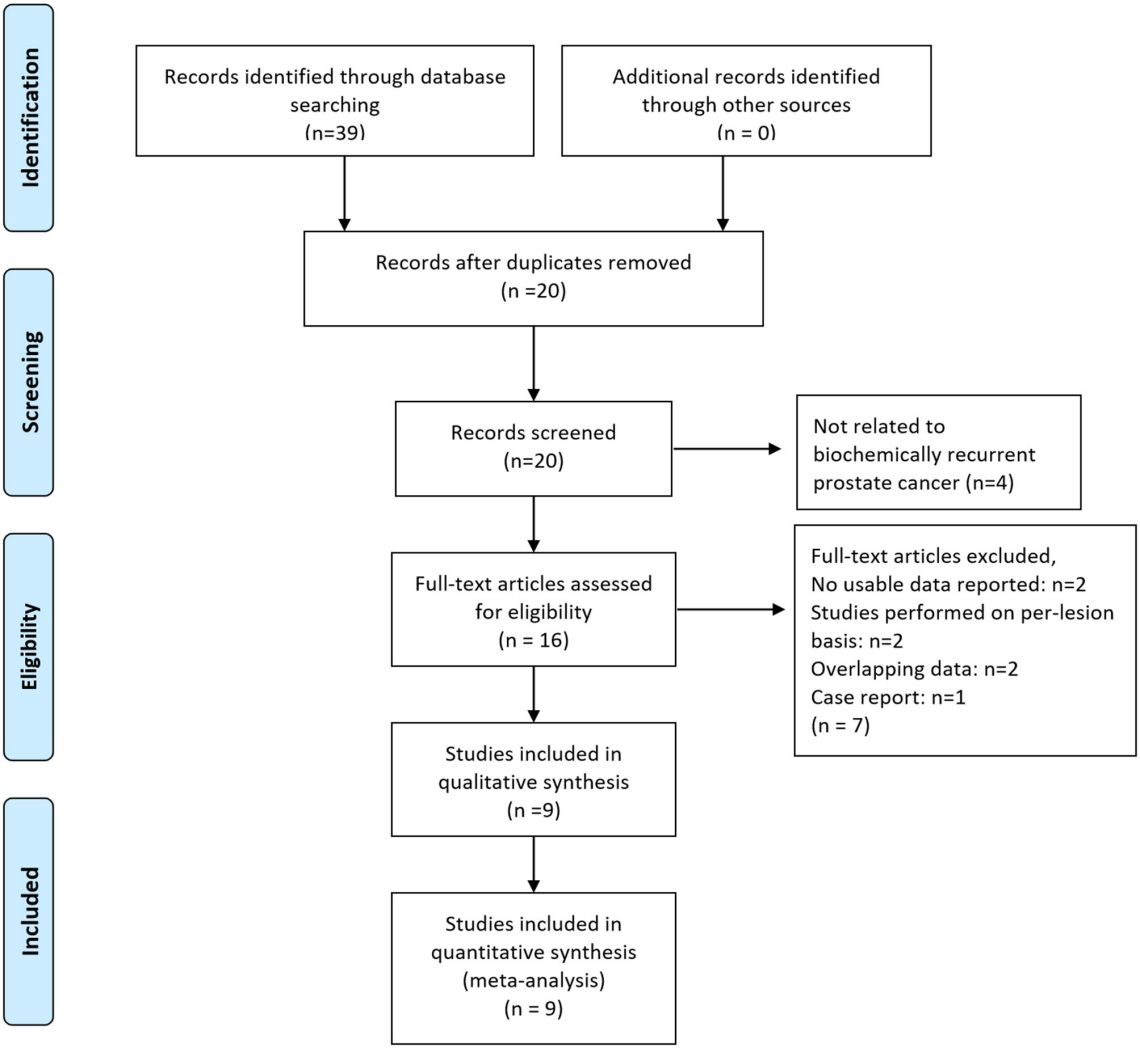
Preferred reporting items for systematic reviews and meta-analyses (PRISMA) 2009 flow diagram. From Moher et al. (28).

As shown in the PRISMA flow diagram (Figure 1), nine studies containing 844 patients were finally included for further meta-analysis (31–39). The QUADAS-2 shows that the quality of all articles included was not completely satisfactory because some articles did not detail patient selection information (Supplementary Materials 1.1, 1.2).

Basic characteristics and technical aspects of the involved studies were summarized in Tables 1, 2, respectively. On the whole, all included studies shared a similar type of patients evaluated and detailed techniques of 18F-DCFPyL PET/CT. We also noticed that the mean/median PSA levels of the included BRPCa patients before 18F-DCFPyL PET/CT scanning could be obviously divided into two groups: high-PSA level group (25, 28, 29) and low-PSA level group (26, 27, 30–33).

**Table 1:** Summary of the included studies.

**References**	**Study period**	**Country**	**Study design**	**Type of patients evaluated**	**No. of patients**	**Mean/median age**	**Gleason Score**	**Mean/median PSA values before PET/CT (ng/ml)**	**Mean/median PSA doubling time before PET/CT (months)**
Markowski et al. (31)	NA	USA	Prospective single-center	Patients with BRPCa previously treated with RP.	108	Median: 67 (61–71)	Gleason ≤ 7 (70.4%) Gleason ≥8 (26.8%) Unknown (2.8%)	Median: 0.7 (0.3–1.8)	NA
Hong et al. (32)	2018–2019	USA	Prospective single-center	Patients with BRPCa previously treated with RP (58.3%) or RT (41.7%) with or without ADT.	72	Mean: 71.5 ± 7.2	Gleason ≤ 6 (8%) Gleason 7 (51%) Gleason ≥8 (40%)	Median: 3.0 (0.23–698.4) Mean: 15.8 ± 58.2	NA
Wei et al. (33)	2017–2018	Canada	Prospective multicenter	Patients with BRPCa previously treated with RT (100.0%) with or without additional ADT.	79	Median: 75 (51–88)	Gleason ≤ 6 (29.1%) Gleason 7 (65.9%) Gleason ≥8 (5.1%)	Median: 4.8 (2.1–69)	Median: 14.4 (1.9–48.6)
Jansen et al. (34)	2018–2019	Netherlands	Retrospective single-center	Patients with BRPCa previously treated with RP without ADT.	24	Median: 67 (61–77)	NA	Median: 0.7 (0.4–1.9)	NA
Rowe et al. (35)	NA	USA	Prospective single-center	Patients with BRPCa previously treated with RP.	31	Median: 63 (45–74)	NA	Median: 0.4 (0.2–28.3)	NA
Mena et al. (36)	NA	USA	Prospective single-center	Patients with BRPCa previously treated with RP (42.2%) or RT (30.0%) or RP+RT (27.8%) without ADT.	90	Median: 66 (50–81)	Gleason ≤ 6 (14.4%) Gleason 7 (35.6%) Gleason≥8 (50.0%)	Median: 2.5 (0.21–35.5)	Median: 7.0 (0.9–75.2)
Rousseau et al. (37)	NA	Canada	Prospective single-center	Patients with BRPCa previously treated with RP (72.3%) or RT (34.6%) with or without additional ADT.	130	Mean: 69.1 ± 6.5	Gleason ≤ 6 (13%) Gleason 7 (50%) Gleason≥8 (37%)	Mean: 5.2 ± 6.5	Mean: 12.2 ± 11.8
Wondergem et al. (38)	2016–2018	Netherlands	Retrospective multicenter	Patients with BRPCa previously treated with RP or RT with or without ADT.	248	Median: 71 (67–75)	Gleason ≤ 6 (13%) Gleason 7 (39%) Gleason≥8 (34%) Unknown (14%)	NA	Median: 6 (3–12)
Dietlein et al. (39)	NA	Germany	Retrospective single-center	Patients with BRPCa previously treated with RP (61%) or RT (39%).	62	Mean: 68	Gleason ≤ 6 (7%) Gleason 7 (56%) Gleason≥8 (37%)	Mean: 3.2	NA

*NA, not available; BRPCa, biochemically recurrent prostate cancer; RP, radical prostatectomy; RT = radiation therapy; ADT, androgen deprivation therapy; PET/CT, positron emission tomography/computed tomography*.

**Table 2:** Technical aspects of the included studies.

**References**	**Radiotracer**	**Hybrid imaging modality**	**Fasting before radiotracer injection**	**Mean/median radiotracer injected activity**	**Time between radiotracer injection and image acquisition**	**Image analysis**	**Other imaging performed for comparison**
Markowski et al. (31)	18F-DCFPyL	NA	NA	333 MBq	60 min	Visual	NA
Hong et al. (32)	18F-DCFPyL	PET/CT with low-dose CT	NA	338.8 ± 25.3 (270.1–370) MBq	74.4 ± 10.4 min	Visual	CT, mpMRI, bone scan, ^18^F-NaF PET/CT, and ^18^F-fluciclovine PET/CT
Wei et al. (33)	18F-DCFPyL	PET/CT with low-dose CT	NA	Mean: 333 MBq (299.7–366.3) MBq	60 ± 10 min	Visual	CT, mpMRI, and bone scan
Jansen et al. (34)	18F-DCFPyL	PET/CT with low-dose CT	NA	Median: 314.4 MBq (257.7–328.6) MBq	120 ± 21 min	Visual	NA
Rowe et al. (35)	18F-DCFPyL	NA	NA	No more than 333 MBq	60 min	Visual and semiquantitative (SUVmax)	NA
Mena et al. (36)	18F-DCFPyL	PET/CT with low-dose CT	NA	Mean: 299.3 MBq (229.4–325.6) MBq	120 min	Visual and semiquantitative (SUVmax, TV, VOI)	NA
Rousseau et al. (37)	18F-DCFPyL	PET/CT with low-dose CT	Yes (at least 4 h)	369.2 ± 47.2 (237–47 ×4) MBq	120 min	Visual and semiquantitative (SUVmax, SUVpeak, SUL, TLG, SUVratio)	NA
Wondergem et al. (38)	18F-DCFPyL	PET/CT with low-dose CT or contrast-enhanced CT	NA	Median: 311 MBq (284–325) MBq	120 min	Visual	NA
Dietlein et al. (39)	18F-DCFPyL	PET/CT with low-dose CT	Yes (at least 4 h)	269.8 ± 81.8 MBq	120 min	Visual and semiquantitative (SUVmax)	^68^Ga-PSMA-11 PET/CT

*NA, not available; PET/CT, positron emission tomography/computed tomography; mpMRI, multiparametric magnetic resonance imaging; 18F-DCFPyL, 2-(3-{1-carboxy-5-[(6-[18F] fluoro-pyridine-3-carbonyl)-amino]-pentyl}-ureido)-pentanedioic acid; MBq, megabecquerel*.

The main findings of the included studies about 18F-DCFPyL PET/CT in BRPCa patients are shown in Table 3. The pooled DR was 88.8% for PSA ≥ 0.5 ng/ml (95% CI: 86.2–91.3%) and 47.2% for PSA < 0.5 ng/ml (95% CI: 32.6–61.8%). The pooled DRs in local recurrence, regional lymph node recurrence, distant lymph node recurrence, bone, and organ were 36.6% (95% CI: 33.1–40.2%), 45.8% (95% CI: 42.1–49.6%), 19.3% (95% CI: 16.2–22.3%), 20.5% (95% CI: 17.3–23.6%), and 4.2% (95% CI: 2.6–6.0%), respectively.

**Table 3:** Main findings of the included studies about 18F-DCFPyL PET/CT in BRPCa patients.

**References**	**Overall DR on a per patient-based analysis**	**DR in patients with PSA < 0.5 ng/ml**	**DR in patients with PSA ≥ 0.5 ng/ml**	**DR in patients with PSA between 0.5 and 1 ng/ml**	**DR in patients with PSA between 1 and 2 ng/ml**	**DR in patients with PSA ≥ 2 ng/ml**	**Local recurrence**	**Regional lymph node recurrence**	**Distant lymph node recurrence**	**Bone**	**Organ**
Markowski et al. (31)	82/108 (75.9%)	26/46 (56.5%)	56/62 (90.3%)	NA	NA	NA	NA	NA	NA	NA	NA
Hong et al. (32)	61/72 (84.7%)	4/8 (50%)	57/64 (89.0%)	9/13 (69%)	5/5 (100%)	43/46 (93.5%)	22/72 (31%)	34/72 (48%)	20/72 (28%)	33/72 (46%)	11/72 (16%)
Wei et al. (33)	69/79 (87.0%)	NA	NA	NA	NA	NA	54/79 (68%)	21/79 (27%)	14/79 (18%)	NA	NA
Jansen et al. (34)	16/24 (66.7%)	NA	NA	NA	NA	NA	NA	NA	NA	NA	NA
Rowe et al. (35)	21/31 (67.7%)	NA	NA	NA	NA	NA	8/31 (25.8%)	14/31 (45.1%)	2/31 (6.5%)	2/31 (6.5%)	0/31 (0%)
Mena et al. (36)	70/90 (77.8%)	10/21 (47.6%)	60/69 (90%)	5/10 (50%)	8/9 (88.9%)	47/50 (94%)	29/90 (32.2%)	39/90 (43.3%)	17/90 (18.8%)	9/90 (10.0%)	5/90 (5.5%)
Rousseau et al. (37)	110/130 (84.6%)	3/5 (60%)	107/125 (85.6%)	18/23 (78.3%)	18/25 (72%)	71/77 (92.2%)	35/130 (26.9%)	57/130 (43.8%)	32/130 (24.6%)	26/130 (20.0%)	3/130 (2.3%)
Wondergem et al. (38)	214/248 (86.3%)	17/29 (59%)	197/217 (90.8%)	20/29 (69%)	35/41 (85%)	142/149 (95%)	92/248 (37.1%)	136/248 (54%)	49/248 (19.8%)	73/248 (29.4%)	12/248 (4.8%)
Dietlein et al. (39)	46/62 (74.2%)	1/8 (12.5%)	45/54 (83.3%)	NA	NA	NA	NA	NA	NA	NA	NA
Pooled values (95% confidence interval)	80.2% (75.6–84.7%)	47.2% (32.6–61.8%)	88.8% (86.2–91.3%)	70.3% (60.2–80.5%)	82.9% (74.5–91.3%)	94.3% (91.8–96.9%)	36.6% (33.1–40.2%)	45.8% (42.1–49.6%)	19.3% (16.2–22.3%)	20.5% (17.3–23.6%)	4.2% (2.6–6.0%)

*NA, not available; DR, detection rate; PSA, prostate-specific antigen; BRPCa, biochemically recurrent prostate cancer; 18F-DCFPyL, 2-(3-{1-carboxy-5-[(6-[18F] fluoro-pyridine-3-carbonyl)-amino]-pentyl}-ureido)-pentanedioic acid; PET/CT, positron emission tomography/computed tomography*.

### Quantitative Synthesis

**Figure 2:**
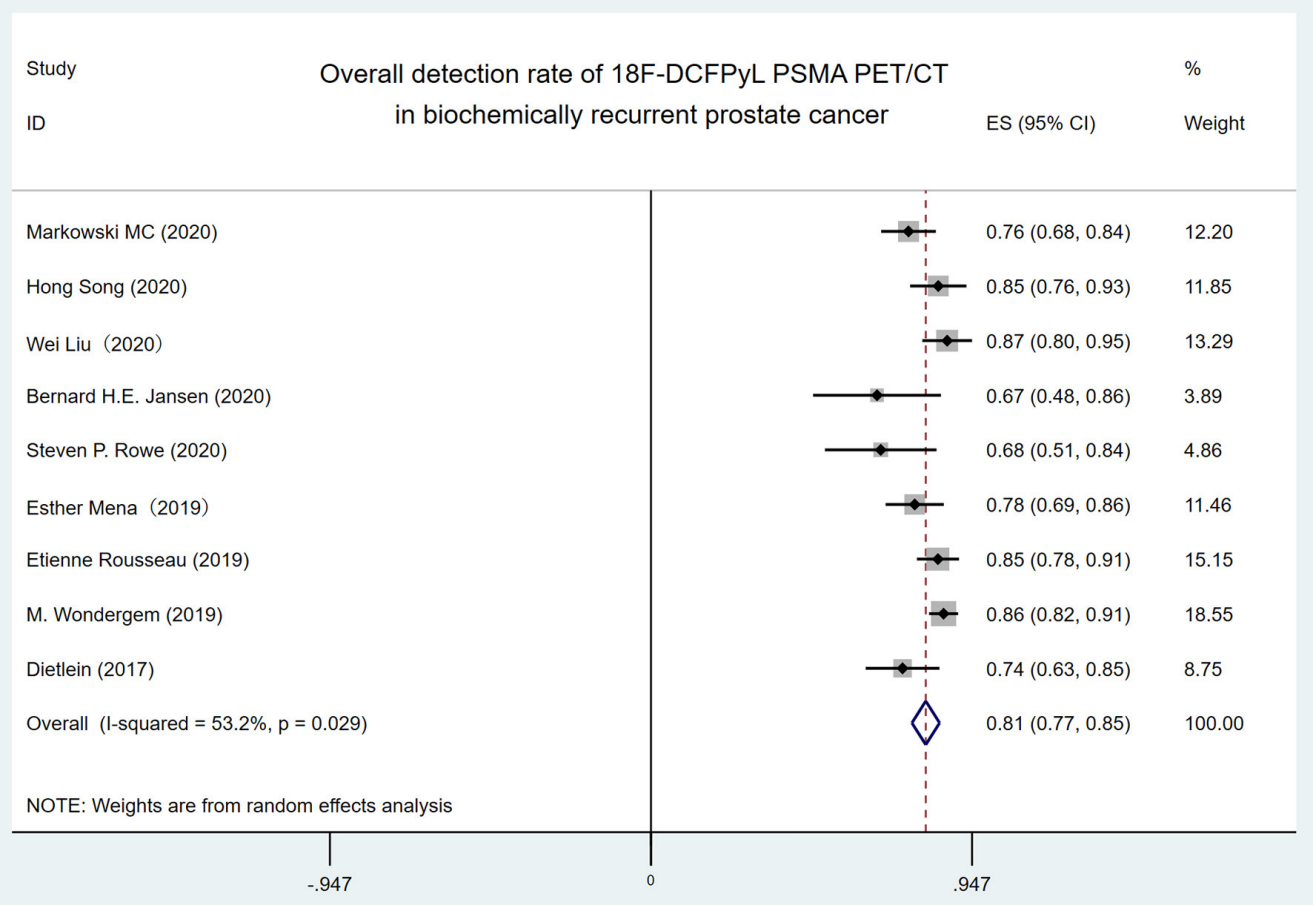
Forest plot for overall detection rate of 18F-DCFPyL PSMA PET/CT in biochemically recurrent prostate cancer. 18F-DCFPyL, 2-(3-{1-carboxy-5-[(6-[18F] fluoro-pyridine-3-carbonyl)-amino]-pentyl}-ureido)-pentanedioic acid; PSMA, prostate-specific membrane antigen; PET/CT, positron emission tomography/computed tomography.

The forest plot of the overall DR of 18F-DCFPyL PET/CT in BRPCa was shown in Figure 2. The random effect model demonstrated that the pooled overall DR of 18F-DCFPyL PET/CT in BRPCa was 81% (95% CI: 76.9–85.1%). For *I*^2^ = 53.2%, high heterogeneity was found.

**Figure 3:**
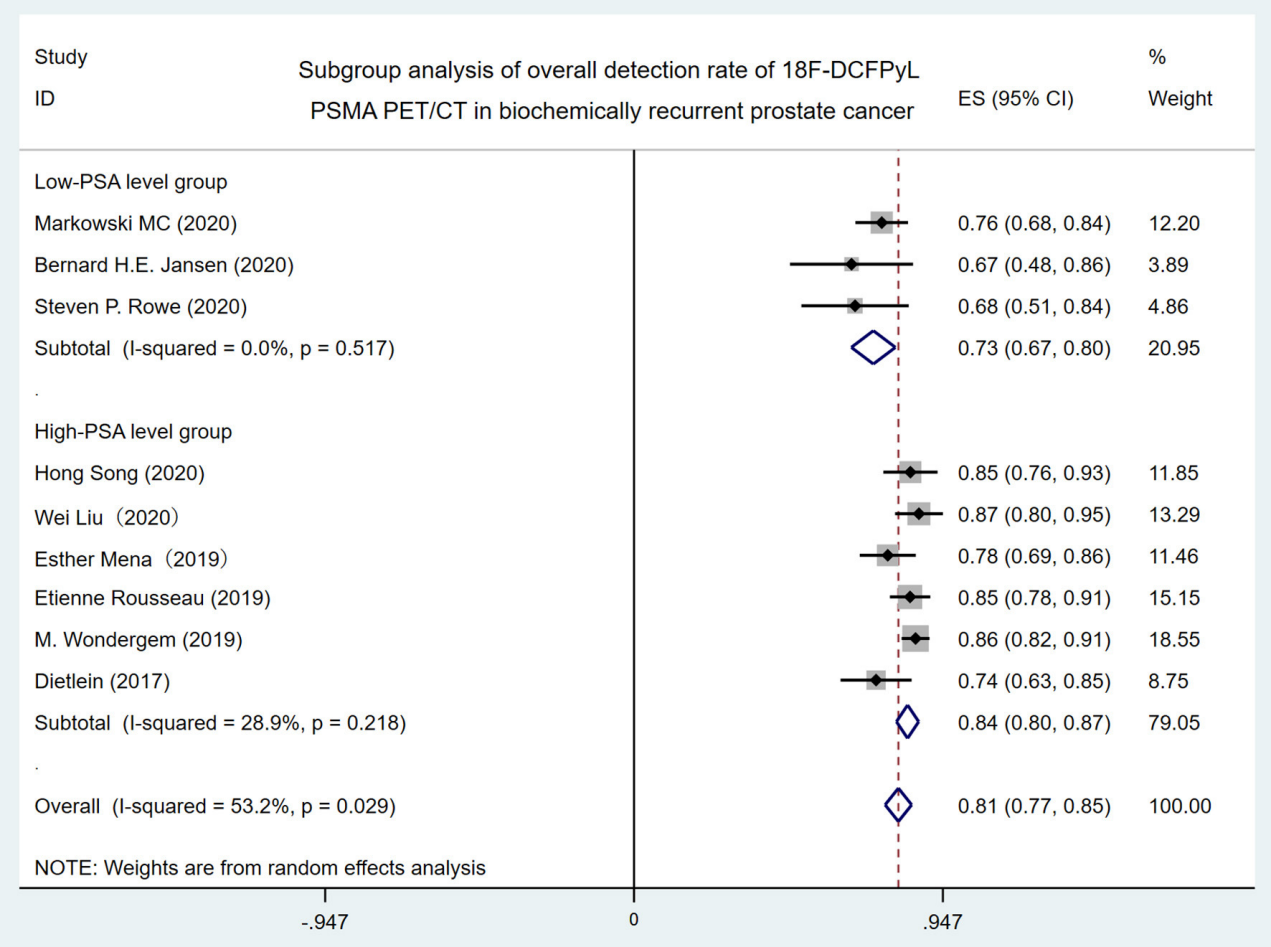
Forest plot for subgroup analysis of overall detection rate of 18F-DCFPyL PSMA PET/CT in biochemically recurrent prostate cancer. 18F-DCFPyL, 2-(3-{1-carboxy-5-[(6-[18F] fluoro-pyridine-3-carbonyl)-amino]-pentyl}-ureido)-pentanedioic acid; PSMA, prostate-specific membrane antigen; PET/CT, positron emission tomography/computed tomography.

In order to identify the source of high heterogeneity, we performed the subgroup analysis (Figure 3) according to the PSA level with a cutoff value of 1 ng/ml before 18F-DCFPyL PET/CT scanning. The results revealed that the pooled overall DR in the low-PSA level group was 73% (95% CI: 67–80%), and the pooled overall DR in the high-PSA level group was 84% (95% CI: 77–85%). For *I*^2^ = 0 and 28.9%, no high heterogeneity was found.

**Figure 4:**
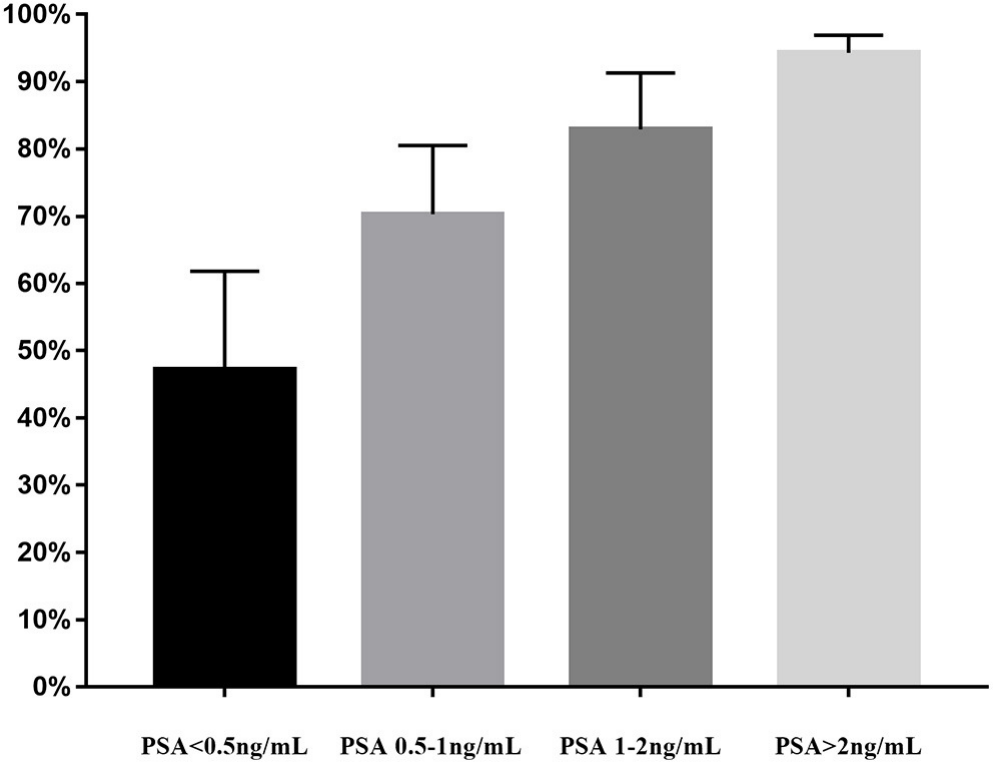
PSA-stratified performance of detection of 18F-DCFPyL PET/CT in biochemically recurrent prostate cancer. PSA, prostate-specific antigen; 18F-DCFPyL, 2-(3-{1-carboxy-5-[(6-[18F] fluoro-pyridine-3-carbonyl)-amino]-pentyl}-ureido)-pentanedioic acid; PSMA, prostate-specific membrane antigen; PET/CT, positron emission tomography/computed tomography.

Lastly, we performed the analysis of detection rate of 18F-DCFPyL PET/CT in BRPCa stratified by different PSA levels (Figure 4). The pooled DR was 47.2% for PSA < 0.5 ng/ml (95% CI: 32.6–61.8%), 70.3% for PSA 0.5–1 ng/ml (95% CI: 60.2–80.5%), 82.9% for PSA 1–2 ng/ml (95% CI: 74.5–91.3%), and 94.3% for PSA >2 ng/ml (95% CI: 91.8–96.9%).

### Publication Bias

We quantified publication bias by the Egger method. Publication bias was found in the overall DR of 18F-DCFPyL PET/CT in BRPCa (*P* = 0.021). The Egger graph was shown in Supplementary Material 2.

## Discussion

Although several meta-analyses have evaluated the diagnostic performance of PET/CT in BRPCa (40–45), to our knowledge, this is the first systematic review that focuses on the role of 18F-DCFPyL PSMA PET/CT in early detection of recurrent lesions in BRPCa patients. Besides, our data include the most up-to-date studies that have been published over the past year. Crocerossa et al. (42) reported that the overall DR of 68Ga-PSMA PET/CT was 72.4%. Fanti et al. (43) reported that the overall DR of 11C-choline PET/CT was 62% (95% CI: 53–71%). Von Eyben and Kairemo (44) reported that the overall DR of 18F-fluorocholine PET/CT was 66%, and our result suggests that the overall DR of 18F-DCFPyL PET/CT is 81% (95% CI: 76.9–85.1%). In conclusion, it shows that 18F-DCFPyL PET/CT has a relatively higher overall DR in BRPCa than other techniques mentioned above, which may enable physicians to make earlier clinical decisions for preventing further metastasis. Nevertheless, the results should be prudentially concluded due to the existence of publication bias (*P* = 0.021).

Of all our nine included studies, Jansen et al. (34) and Rowe et al. (35) reported a relatively low overall DR (66.7 and 67.7%, respectively.) in comparison with Song et al. (32), Liu et al. (33), Rousseau et al. (37), and Wondergem et al. (38) (84.7, 87.0, 84.6, and 86.3%, respectively). This is mainly because the participants in the former two studies had lower PSA levels before 18F-DCFPyL PET/CT (0.7 and 0.4 ng/ml, respectively). Another possible reason is that these two studies did not detail the distribution of Gleason scores of their participants. In addition, prior published studies have shown that PSA doubling time was a strong predictor for developing metastatic disease using conventional imaging (46, 47). However, we did not further analyze the association between PSA doubling time and a positive DR, since there were only four of our included studies (33, 36–38) reporting the specific mean/median PSA doubling time before 18F-DCFPyL PET/CT. Lastly, Markowski et al. (31), Jansen et al. (34), and Rowe et al. (35) included patients who were only treated with RP previously. Liu et al. (33) included patients who were only treated with RT previously. Other five studies included patients who were treated with RP or RT previously. Since the definition of BCR after RP is different from that after RT, patients who received different types of therapies could also possibly affect the DR of 18F-DCFPyL PET/CT.

High heterogeneity (*I*^2^ = 53.2%) was found in the overall DR of 18F-DCFPyL PET/CT in BRPCa patients. When the included studies were classified by the mean/median PSA level with 1 ng/ml before 18F-DCFPyL PET/CT scanning in subgroup analysis, the *I*^2^ decreased from 53.2 to 0% and 28.9%, respectively (Figure 3). Thus, it shows that the 18F-DCFPyL may exhibit highly consistent sensitivity in BRPCa with higher PSA levels, which is in accordance with our clinical experience.

However, we did not perform a detailed analysis about the DR of 18F-DCFPyL PET/CT in local recurrence, regional lymph node recurrence, distant lymph node recurrence, bone, and organ. The reasons are as follows. For one thing, since the place for cancer to metastasize varies between individuals in clinical practice, it is much more reasonable to evaluate DR in recurrent sites on a per-lesion basis. However, all included studies in our meta-analysis only reported DR in recurrent sites on a per-person basis but not on a per-lesion basis. For another, due to the lack of specific corresponding relation between PSA levels and DR in different recurrent sites in our included studies, we could not conduct the analysis of DR in different recurrent sites stratified by different PSA levels.

Treglia et al. (40) previously reported that 18F-labeled PSMA PET/CT had a better DR in BRPCa patients while PSA level was rising. Similarly, our meta-analysis also illustrated a trend that 18F-DCFPyL PSMA PET/CT may exhibit improved sensitivity in BRPCa with increased PSA levels (Figure 4). For PSA 1–2 ng/ml and PSA > 2 ng/ml, the overall DR of 18F-DCFPyL PET/CT significantly rose up to 82.9% (95% CI: 74.5–91.3%) and 94.3% (95% CI: 91.8–96.9%), respectively. By contrast, for PSA < 0.5 ng/ml and 0.5–1 ng/ml, the overall DRs were only 47.2% (95% CI: 32.6–61.8%) and 70.3% (95% CI: 60.2–80.5%), respectively.

Thus, an optimal PSA threshold that would justify 18F-DCFPyL PET/CT imaging is necessary to be established. For 11C-choline PET/CT, Castellucci et al. (48) showed an optimal cutoff point for trigger PSA of 2.43 ng/ml. For 68Ga-PSMA PET/CT, Hope et al. (49) reported an optimal PSA threshold of 1.5 ng/ml. For 18F fluorocholine PET/CT, Gauvin et al. (50) suggested that a trigger PSA of 2.6 ng/ml had a sensitivity of 84% and specificity of 65% for a positive scan. However, the optimal PSA threshold for 18F-DCFPyL PET-CT has not been suggested yet. Our study revealed that the pooled overall DR of 18F-DCFPyL PET/CT was 88.8% for PSA ≥ 0.5 ng/ml (95% CI: 86.2–91.3%) and 47.2% for PSA < 0.5 ng/ml (95% CI: 32.6–61.8%). Basing on the above results and considering the high cost of PET/CT scans, we assumed that a PSA threshold of 0.5 ng/ml for 18F-DCFPyL PET/CT might be reasonable and cost-effective. That means 18F-DCFPyL PET/CT could detect recurrent sites in BCR patients at lower PSA levels compared with other targeted radiotracers. Moreover, its higher sensitivity enables urologists to tailor earlier salvage procedures or medical treatment and potentially influence outcome. Nevertheless, our results were based on only nine studies published in recent years. Further large-scale studies are warranted to prove its significant advantages and clinical values.

Over the past decades, a variety of targeted radiotracers including 11C/18F-choline, 18F-fluciclovine, 68Ga-PSMA, and 18F-PSMA have been proposed. In 2012, choline was approved by the U.S. Food and Drug Administration (FDA) as an imaging agent to be used to detect PCa during PET imaging. However, choline is accumulating not only in malignant tissues but also in inflammatory diseases. It means that choline PET/CT imaging may be limited for the differentiation of malignant and benign lesions, which is particularly important in lymph nodes (51). The use of fluciclovine was approved by the U.S. FDA in May of 2016, and its rational biodistribution and slow renal excretion make it suitable for imaging of suspected PCa recurrence following treatment (52). Similarly, a major disadvantage of the 18F-fluciclovine PET tracer is its nonspecific uptake by benign inflammatory prostatic tissue (53). In addition, the review of the data demonstrated lower detection rates of 18F-choline, 18F-fluciclovine for each respective PSA cohort (54). Thus, PCa-specific PET/CT radiotracers such as 68Ga-PSMA and 18F-PSMA seem to show superiority and provide new insight into the early patterns of disease spread. Until now, 68Ga-PSMA is the most commonly used radiotracer in clinical practice (55). It is the first drug for PET imaging of PSMA-positive lesions in men with PCa approved by the U.S. FDA in December of 2020 whether or not the cancer has spread to other parts of the body (56). However, recent years have witnessed the beginning of a shift from 68Ga to 18F-labeled compounds (57). 18F-DCFPyL, the second-generation 18F-labeled PSMA radiotracers, has also received increasing recognition recently since it was proposed (58). Because of the lower positron emission energy of 18F-DCFPyL, the distance to decelerate the positron in human tissue is much shorter in comparison with 68Ga-PSMA, resulting in a higher image resolution. Furthermore, production volume and a longer half-life offer practical advantages over 68Ga-PSMA (10). Given the above diagnostic advantages, the role of 18F-DCFPyL PET/CT may extend beyond BRPCa to the initial staging of high-risk PCa in the future.

The study has several limitations. Firstly, none of the included studies has complete histologic validation. In a lack of histological validation, it cannot be excluded that some lesions detected by 18F-DCFPyL PET/CT may represent false-positive findings. Secondly, we did not further analyze the pooled DR of 18F-DCFPyL PET/CT based on recurrent sites. Thirdly, patients who receive ADT or not after RP or RT may share different BCR progression, PSA levels, and metastatic sites. All these conditions could possibly affect the DR of 18F-DCFPyL PSMA PET/CT. However, due to the lack of this specific information in our included studies, we could not conduct the subgroup analysis of overall DR divided by patients with or without ADT and it might be a confounder in our study. Lastly, publication bias was observed in our study. Large-scale and well-designed studies are warranted for a valid conclusion.

## Conclusion

Despite some limitations, our meta-analysis revealed that 18F-DCFPyL PET/CT has a relatively high DR in BRPCa. To prove our results, future large-scale and well-designed studies are needed to provide more powerful evidence.

## Data Availability Statement

The original contributions presented in the study are included in the article/Supplementary Material, further inquiries can be directed to the corresponding author/s.

## Author Contributions

JS performed the experiments, analyzed the data, contributed analysis tools, and wrote the paper. YL performed the experiments and prepared figures and/or tables. XW and JO analyzed the data and prepared figures and/or tables. ZL and YH conceived and designed the experiments and reviewed drafts of the paper. All authors contributed to the article and approved the submitted version.

## Conflict of Interest

The authors declare that the research was conducted in the absence of any commercial or financial relationships that could be construed as a potential conflict of interest.

## Abbreviations

PSMA: prostate-specific membrane antigen
PET/CT: positron emission tomography/computed tomography
BRPCa: biochemically recurrent prostate cancer
CI: confidence interval
DR: detection rate
PSA: prostate-specific antigen
PCa: prostate cancer
BCR: biochemical recurrence
RP: radical prostatectomy
EBRT: external beam radiation therapy
RT: radiation therapy
PRISMA: Preferred Reporting Items for Systematic Reviews and Meta-analyses
QUADAS-2: quality assessment of diagnostic accuracy studies
18F-DCFPyL: 2-(3-{1-carboxy-5-[(6-[18F] fluoro-pyridine-3-carbonyl)-amino]-pentyl}-ureido)-pentanedioic acid.

## References

[1] Siegel RL Miller KD Jemal A. Cancer statistics, 2020. CA Cancer J Clin. (2020) 70:7–30. 10.3322/caac.21590 31912902

[2] Mottet N Bellmunt J Bolla M Briers E Cumberbatch MG De Santis M. EAU-ESTRO-SIOG guidelines on prostate cancer. Part 1: screening, diagnosis, and local treatment with curative intent. Eur Urol. (2017) 71:618–29. 10.1016/j.eururo.2016.08.003 27568654

[3] Cookson MS Aus G Burnett AL Canby-Hagino ED D'Amico AV Dmochowski RR. Variation in the definition of biochemical recurrence in patients treated for localized prostate cancer: the American Urological Association Prostate Guidelines for Localized Prostate Cancer Update Panel report and recommendations for a standard in the reporting of surgical outcomes. J Urol. (2007) 177:540–5. 10.1016/j.juro.2006.10.097 17222629

[4] Roach M 3rd Hanks G Thames H Jr Schellhammer P Shipley WU. Defining biochemical failure following radiotherapy with or without hormonal therapy in men with clinically localized prostate cancer: recommendations of the RTOG-ASTRO Phoenix Consensus Conference. Int J Radiat Oncol Biol Phys. (2006) 65:965–74. 10.1016/j.ijrobp.2006.04.029 16798415

[5] Han M Partin AW Zahurak M Piantadosi S Epstein JI Walsh PC. Biochemical (prostate specific antigen) recurrence probability following radical prostatectomy for clinically localized prostate cancer. J Urol. (2003) 169:517–23. 10.1016/S0022-5347(05)63946-8 12544300

[6] Freedland SJ Humphreys EB Mangold LA Eisenberger M Dorey FJ Walsh PC. Risk of prostate cancer-specific mortality following biochemical recurrence after radical prostatectomy. JAMA. (2005) 294:433–9. 10.1001/jama.294.4.433 16046649

[7] Roehl KA Han M Ramos CG Antenor JA Catalona WJ. Cancer progression and survival rates following anatomical radical retropubic prostatectomy in 3,478 consecutive patients: long-term results. J Urol. (2004) 172:910–4. 10.1097/01.ju.0000134888.22332.bb 15310996

[8] Kupelian PA Mahadevan A Reddy CA Reuther AM Klein EA. Use of different definitions of biochemical failure after external beam radiotherapy changes conclusions about relative treatment efficacy for localized prostate cancer. Urology. (2006) 68:593–8. 10.1016/j.urology.2006.03.075 16979731

[9] Lazarev S Thompson MR Stone NN Stock RG. Low-dose-rate brachytherapy for prostate cancer: outcomes at >10 years of follow-up. BJU Int. (2018) 121:781–90. 10.1111/bju.14122 29319928

[10] Spratt DE McHugh DJ Morris MJ Morgans AK. Management of biochemically recurrent prostate cancer: ensuring the right treatment of the right patient at the right time. Am Soc Clin Oncol Educ Book. (2018) 38:355–62. 10.1200/EDBK_200319 30231323

[11] Rowe SP Mana-Ay M Javadi MS Szabo Z Leal JP Pomper MG. PSMA-based detection of prostate cancer bone lesions with (1)(8)F-DCFPyL PET/CT: a sensitive alternative to ((9)(9)m)Tc-MDP Bone Scan and Na(1)(8)F PET/CT? Clin Genitourinary Cancer. (2016) 14:e115–8. 10.1016/j.clgc.2015.09.011 PMC4698229 26603549

[12] Rajasekaran AK Anilkumar G Christiansen JJ. Is prostate-specific membrane antigen a multifunctional protein? Am J Physiol Cell Physiol. (2005) 288:C975–81. 10.1152/ajpcell.00506.2004 15840561

[13] Maurer T Eiber M Schwaiger M Gschwend JE. Current use of PSMA-PET in prostate cancer management. Nat Rev Urol. (2016) 13:226–35. 10.1038/nrurol.2016.26 26902337

[14] Eapen RS Nzenza TC Murphy DG Hofman MS Cooperberg M Lawrentschuk N. PSMA PET applications in the prostate cancer journey: from diagnosis to theranostics. World J Urol. (2019) 37:1255–61. 10.1007/s00345-018-2524-z 30374609

[15] Thoma C. PSMA PET-CT outperforms conventional imaging in high-risk prostate cancer. Nat Rev Urol. (2020) 17:319. 10.1038/s41585-020-0330-z 32371916

[16] Diao W Cao Y Su D Jia Z. Impact of (68) Gallium prostate-specific membrane antigen tracers on the management of patients with prostate cancer who experience biochemical recurrence. BJU Int. (2021) 127:153–63. 10.1111/bju.15257 32979229

[17] Ghodsirad MA Pirayesh E Akbarian R Javanmard B Kaghazchi F Tavakoli M. Diagnostic utility of lutetium-177 (Lu 177) prostate-specific membrane antigen (PSMA) scintigraphy in prostate cancer patients with PSA rise and negative conventional imaging. Urol J. (2020) 17:374–8. 10.22037/uj.v0i0.5451 32281092

[18] Mirzaei S Mohammed F Zandieh S. Theranostics of metastatic prostate cancer applying 64Cu/18F/68Ga PSMA PET-CT and 177Lu radiopharmaceuticals. Curr Radiopharm. (2020). 10.2174/1874471013666200908122845. [Epub ahead of print]. 32900357

[19] Paymani Z Rohringer T Vali R Loidl W Alemohammad N Geinitz H. Diagnostic performance of [(18)F]Fluorocholine and [(68)Ga]Ga-PSMA PET/CT in prostate cancer: a comparative study. J Clin Med. (2020) 9:2308. 10.3390/jcm9072308 PMC7408886 32708097

[20] Kelly JM Ponnala S Amor-Coarasa A Zia NA Nikolopoulou A Williams C. Preclinical evaluation of a high-affinity sarcophagine-containing PSMA ligand for (64)Cu/(67)Cu-based theranostics in prostate cancer. Mol Pharm. (2020) 17:1954–62. 10.1021/acs.molpharmaceut.0c00060 32286841

[21] Eiber M Maurer T Souvatzoglou M Beer AJ Ruffani A Haller B. Evaluation of hybrid (6)(8)Ga-PSMA ligand PET/CT in 248 patients with biochemical recurrence after radical prostatectomy. J Nucl Med. (2015) 56:668–74. 10.2967/jnumed.115.154153 25791990

[22] Bluemel C Krebs M Polat B Linke F Eiber M Samnick S. 68Ga-PSMA-PET/CT in patients with biochemical prostate cancer recurrence negative 18F-choline-PET/CT. Clin Nucl Med. (2016) 41:515–21. 10.1097/RLU.0000000000001197 26975008 PMC5006491

[23] Afshar-Oromieh A Malcher A Eder M Eisenhut M Linhart HG Hadaschik BA. PET imaging with a [68Ga]gallium-labelled PSMA ligand for the diagnosis of prostate cancer: biodistribution in humans and first evaluation of tumour lesions. Eur J Nucl Med Mol Imaging. (2013) 40:486–95. 10.1007/s00259-012-2298-2 23179945

[24] Ferreira G Iravani A Hofman MS Hicks RJ. Intra-individual comparison of (68)Ga-PSMA-11 and (18)F-DCFPyL normal-organ biodistribution. Cancer Imaging. (2019) 19:23. 10.1186/s40644-019-0211-y 31092293 PMC6521415

[25] Kelly J Amor-Coarasa A Nikolopoulou A Kim D Williams C Jr. Synthesis and pre-clinical evaluation of a new class of high-affinity (18)F-labeled PSMA ligands for detection of prostate cancer by PET imaging. Eur J Nucl Med Mol Imaging. (2017) 44:647–61. 10.1007/s00259-016-3556-5 27847991 PMC5323493

[26] Alexander Drzezga CK Matthias Schmidt Georg Kuhnert Boris Zlatopolskiy Bernd Neumaier Markus Dietlein. Application of 18F-labeled PSMA-imaging using [18F]DCFPyL at very low PSA-values may allow curative treatment in recurrent prostate cancer. J Nucl Med. (2016) 57(Suppl. 2). Available online at: https://jnm.snmjournals.org/content/57/supplement_2/561

[27] Morris MJ Pouliot F Saperstein L Rowe SP Gorin MA Josephson DY. A phase III, multicenter study to assess the diagnostic performance and clinical impact of 18F-DCFPyL PET/CT in men with suspected recurrence of prostate cancer (CONDOR). J Clin Oncol. (2019) 37(15_Suppl.):TPS5093-TPS. 10.1200/JCO.2019.37.15_suppl.TPS5093

[28] Moher D Liberati A Tetzlaff J Altman DG Group P. Preferred reporting items for systematic reviews and meta-analyses: the PRISMA statement. PLoS Med. (2009) 6:e1000097. 10.1371/journal.pmed.1000097 19621072 PMC2707599

[29] Whiting PF Rutjes AW Westwood ME Mallett S Deeks JJ Reitsma JB. QUADAS-2: a revised tool for the quality assessment of diagnostic accuracy studies. Ann Intern Med. (2011) 155:529–36. 10.7326/0003-4819-155-8-201110180-00009 22007046

[30] Higgins JP Thompson SG Deeks JJ Altman DG. Measuring inconsistency in meta-analyses. BMJ. (2003) 327:557–60. 10.1136/bmj.327.7414.557 12958120 PMC192859

[31] Markowski MC Sedhom R Fu W Gray JCR Eisenberger MA Pomper MG. Prostate specific antigen and prostate specific antigen doubling time predict findings on (18)F-DCFPyL positron emission tomography/computerized tomography in patients with biochemically recurrent prostate cancer. J Urol. (2020) 204:496–502. 10.1097/JU.0000000000001064 32250727 PMC7945885

[32] Song H Harrison C Duan H Guja K Hatami N Franc BL. Prospective evaluation of (18)F-DCFPyL PET/CT in biochemically recurrent prostate cancer in an academic center: a focus on disease localization and changes in management. J Nucl Med. (2020) 61:546–51. 10.2967/jnumed.119.231654 31628216

[33] Liu W Zukotynski K Emmett L Chung HT Chung P Wolfson R. A prospective study of 18F-DCFPyL PSMA PET/CT restaging in recurrent prostate cancer following primary external beam radiotherapy or brachytherapy. Int J Radiat Oncol Biol Phys. (2020) 106:546–55. 10.1016/j.ijrobp.2019.11.001 31730876

[34] Jansen BHE Jansen RW Wondergem M Srbljin S de Klerk JMH Lissenberg-Witte BI. Lesion detection and interobserver agreement with advanced image reconstruction for (18)F-DCFPyL PET/CT in patients with biochemically recurrent prostate cancer. J Nucl Med. (2020) 61:210–6. 10.2967/jnumed.118.222513 31481580

[35] Rowe SP Campbell SP Mana-Ay M Szabo Z Allaf ME Pienta KJ. Prospective evaluation of PSMA-targeted (18)F-DCFPyL PET/CT in men with biochemical failure after radical prostatectomy for prostate cancer. J Nucl Med. (2020) 61:58–61. 10.2967/jnumed.119.226514 31201249 PMC6954467

[36] Mena E Lindenberg ML Turkbey IB Shih JH Harmon SA Lim I. (18)F-DCFPyL PET/CT imaging in patients with biochemically recurrent prostate cancer after primary local therapy. J Nucl Med. (2020) 61:881–9. 10.2967/jnumed.119.234799 31676732 PMC9374042

[37] Rousseau E Wilson D Lacroix-Poisson F Krauze A Chi K Gleave M. A prospective study on (18)F-DCFPyL PSMA PET/CT imaging in biochemical recurrence of prostate cancer. J Nucl Med. (2019) 60:1587–93. 10.2967/jnumed.119.226381 30979820 PMC6836862

[38] Wondergem M Jansen BHE van der Zant FM van der Sluis TM Knol RJJ van Kalmthout LWM. Early lesion detection with (18)F-DCFPyL PET/CT in 248 patients with biochemically recurrent prostate cancer. Eur J Nucl Med Mol Imaging. (2019) 46:1911–8. 10.1007/s00259-019-04385-6 31230088 PMC6647179

[39] Dietlein F Kobe C Neubauer S Schmidt M Stockter S Fischer T. PSA-stratified performance of (18)F- and (68)Ga-PSMA PET in patients with biochemical recurrence of prostate cancer. J Nucl Med. (2017) 58:947–52. 10.2967/jnumed.116.185538 27908968

[40] Treglia G Annunziata S Pizzuto DA Giovanella L Prior JO Ceriani L. Detection rate of (18)F-labeled PSMA PET/CT in biochemical recurrent prostate cancer: a systematic review and a meta-analysis. Cancers. (2019) 11:710. 10.3390/cancers11050710 PMC6562935 31126071

[41] Hope TA Goodman JZ Allen IE Calais J Fendler WP Carroll PR. Metaanalysis of (68)Ga-PSMA-11 PET accuracy for the detection of prostate cancer validated by histopathology. J Nucl Med. (2019) 60:786–93. 10.2967/jnumed.118.219501 30530831 PMC6581235

[42] Crocerossa F Marchioni M Novara G Carbonara U Ferro M Russo GI. Detection rate of prostate specific membrane antigen tracers for positron emission tomography/computerized tomography in prostate cancer biochemical recurrence: a systematic review and network meta-analysis. J Urol. (2021) 205:356–69. 10.1097/JU.0000000000001369 32935652

[43] Fanti S Minozzi S Castellucci P Balduzzi S Herrmann K Krause BJ. PET/CT with (11)C-choline for evaluation of prostate cancer patients with biochemical recurrence: meta-analysis and critical review of available data. Eur J Nucl Med Mol Imaging. (2016) 43:55–69. 10.1007/s00259-015-3202-7 26450693

[44] von Eyben FE Kairemo K. Acquisition with (11)C-choline and (18)F-fluorocholine PET/CT for patients with biochemical recurrence of prostate cancer: a systematic review and meta-analysis. Ann Nucl Med. (2016) 30:385–92. 10.1007/s12149-016-1078-7 27173771

[45] Sathianathen NJ Butaney M Konety BR. The utility of PET-based imaging for prostate cancer biochemical recurrence: a systematic review and meta-analysis. World J Urol. (2019) 37:1239–49. 10.1007/s00345-018-2403-7 30003375

[46] Markowski MC Chen Y Feng Z Cullen J Trock BJ Suzman D. PSA doubling time and absolute PSA predict metastasis-free survival in men with biochemically recurrent prostate cancer after radical prostatectomy. Clin Genitourinary Cancer. (2019) 17:470–5 e1. 10.1016/j.clgc.2019.08.002 31530439 PMC9774681

[47] Pound CR Partin AW Eisenberger MA Chan DW Pearson JD Walsh PC. Natural history of progression after PSA elevation following radical prostatectomy. JAMA. (1999) 281:1591–7. 10.1001/jama.281.17.1591 10235151

[48] Castellucci P Fuccio C Nanni C Santi I Rizzello A Lodi F. Influence of trigger PSA and PSA kinetics on 11C-Choline PET/CT detection rate in patients with biochemical relapse after radical prostatectomy. J Nucl Med. (2009) 50:1394–400. 10.2967/jnumed.108.061507 19690023

[49] Hope TA Aggarwal R Chee B Tao D Greene KL Cooperberg MR. Impact of (68)Ga-PSMA-11 PET on management in patients with biochemically recurrent prostate cancer. J Nucl Med. (2017) 58:1956–61. 10.2967/jnumed.117.192476 28522741

[50] Gauvin S Cerantola Y Haberer E Pelsser V Probst S Bladou F. Initial single-centre Canadian experience with 18F-fluoromethylcholine positron emission tomography-computed tomography (18F-FCH PET/CT) for biochemical recurrence in prostate cancer patients initially treated with curative intent. Can Urol Assoc J. (2017) 11:47–52. 10.5489/cuaj.4068 28443145 PMC5403679

[51] Huang SM Yin L Yue JL Li YF Yang Y Lin ZC. Direct comparison of choline PET/CT and MRI in the diagnosis of lymph node metastases in patients with prostate cancer. Medicine. (2018) 97:e13344. 10.1097/MD.0000000000013344 30557983 PMC6320103

[52] Nanni C Schiavina R Rubello D Ambrosini V Brunocilla E Martorana G. The detection of disease relapse after radical treatment for prostate cancer: is anti-3-18F-FACBC PET/CT a promising option? Nucl Med Commun. (2013) 34:831–3. 10.1097/MNM.0b013e3283636eaf 23887227

[53] Schuster DM Nanni C Fanti S Oka S Okudaira H Inoue Y. Anti-1-amino-3-18F-fluorocyclobutane-1-carboxylic acid: physiologic uptake patterns, incidental findings, and variants that may simulate disease. J Nucl Med. (2014) 55:1986–92. 10.2967/jnumed.114.143628 25453047 PMC4844004

[54] Evans JD Jethwa KR Ost P Williams S Kwon ED Lowe VJ. Prostate cancer-specific PET radiotracers: a review on the clinical utility in recurrent disease. Pract Radiat Oncol. (2018) 8:28–39. 10.1016/j.prro.2017.07.011 29037965

[55] Lutje S Heskamp S Cornelissen AS Poeppel TD van den Broek SA Rosenbaum-Krumme S. PSMA ligands for radionuclide imaging and therapy of prostate cancer: clinical status. Theranostics. (2015) 5:1388–401. 10.7150/thno.13348 26681984 PMC4672020

[56] FDA Approves First PSMA-Targeted PET Imaging Drug for Men With Prostate Cancer. (2020). Available online at: https://www.fda.gov/news-events/press-announcements/fda-approves-first-psma-targeted-pet-imaging-drug-men-prostate-cancer

[57] Werner RA Derlin T Lapa C Sheikbahaei S Higuchi T Giesel FL. (18)F-labeled, PSMA-targeted radiotracers: leveraging the advantages of radiofluorination for prostate cancer molecular imaging. Theranostics. (2020) 10:1–16. 10.7150/thno.37894 31903102 PMC6929634

[58] Chen Y Pullambhatla M Foss CA Byun Y Nimmagadda S Senthamizhchelvan S. 2-(3-{1-Carboxy-5-[(6-[18F]fluoro-pyridine-3-carbonyl)-amino]-pentyl}-ureido)-pen tanedioic acid, [18F]DCFPyL, a PSMA-based PET imaging agent for prostate cancer. Clin Cancer Res. (2011) 17:7645–53. 10.1158/1078-0432.CCR-11-1357 22042970 PMC3243762

