# Supplementary material for: Performance of 18F-DCFPyL PET/CT Imaging in Early Detection of Biochemically Recurrent Prostate Cancer: A Systematic Review and Meta-Analysis
Source: Front Oncol. 2021 Apr 26;11:649171. doi: 10.3389/fonc.2021.649171 (PMC8107478; doi:10.3389/fonc.2021.649171)
Supplement: Supplementary file 2 [file Data_Sheet_2.docx]

**Suggested tabular presentation for QUADAS-2 results**

| **Study** | **RISK OF BIAS** | | | | **APPLICABILITY CONCERNS** | | |
| --- | --- | --- | --- | --- | --- | --- | --- |
|  | **PATIENT SELECTION** | **INDEX TEST** | **REFERENCE STANDARD** | **FLOW AND TIMING** | **PATIENT SELECTION** | **INDEX TEST** | **REFERENCE STANDARD** |
| Markowski MC (2020) | ? | ☺ | ☺ | ☺ | ☺ | ? | ☺ |
| Hong Song (2020) | ☺ | ☺ | ☺ | ☺ | ☺ | ☺ | ☺ |
| Wei Liu (2020) | ☺ | ☺ | ☺ | ☺ | ☺ | ? | ☺ |
| Bernard H.E. Jansen (2020) | ☺ | ☺ | ☺ | ☺ | ☺ | ☹ | ☺ |
| Steven P. Rowe (2020) | ? | ☺ | ☺ | ☺ | ☺ | ? | ☺ |
| Esther Mena(2019) | ☺ | ☺ | ☺ | ☺ | ☺ | ☺ | ☺ |
| Etienne Rousseau (2019) | ? | ☺ | ☺ | ☺ | ☺ | ☺ | ☺ |
| M.Wondergem (2019) | ☺ | ☺ | ☺ | ☺ | ☺ | ☺ | ☺ |
| Dietlein (2017) | ? | ☺ | ☺ | ☺ | ☺ | ☹ | ☺ |

☺Low Risk ☹High Risk ? Unclear Risk
