# Supplementary material for: Performance of 18F-DCFPyL PET/CT Imaging in Early Detection of Biochemically Recurrent Prostate Cancer: A Systematic Review and Meta-Analysis
Source: Front Oncol. 2021 Apr 26;11:649171. doi: 10.3389/fonc.2021.649171 (PMC8107478; doi:10.3389/fonc.2021.649171)
Supplement: Supplementary file 3 [file Data_Sheet_3.pdf]

Supplementary 2. Publication bias of detection rate of 18F-DCFPyL PSMA PET/CT in biochemical recurrent prostate cancer.

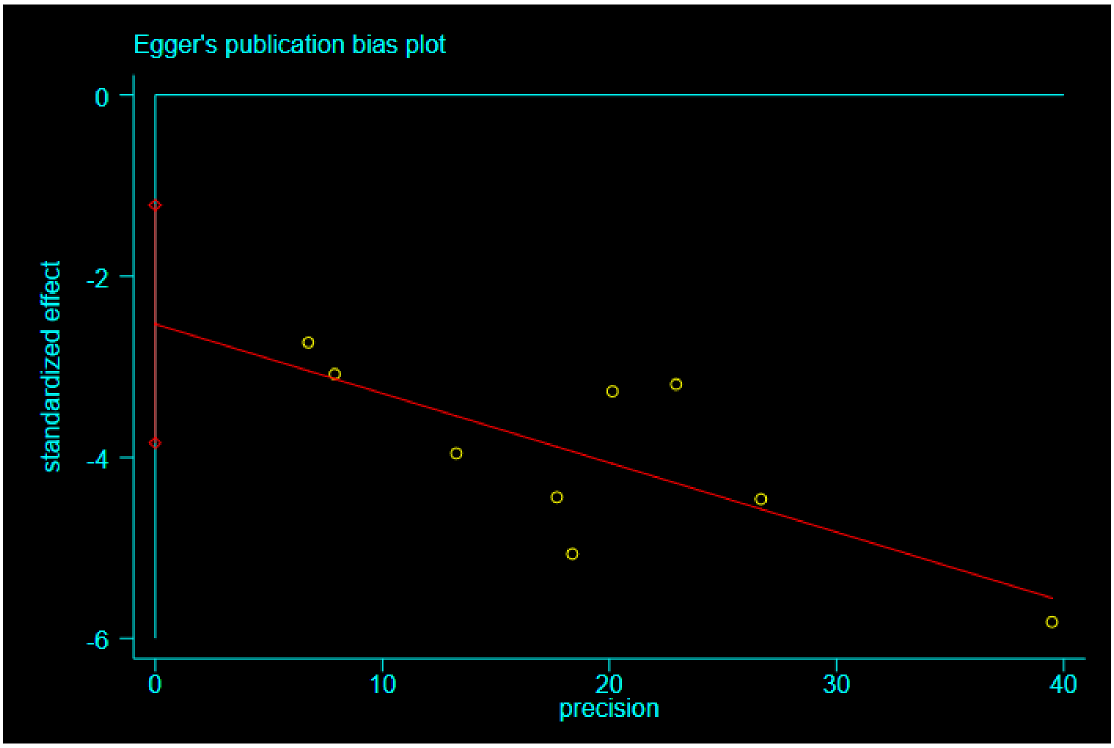

Overall detection rate (P=0.021)
